# Nutritional Characterization and Phenolic Profiling of *Moringa oleifera* Leaves Grown in Chad, Sahrawi Refugee Camps, and Haiti

**DOI:** 10.3390/ijms160818923

**Published:** 2015-08-12

**Authors:** Alessandro Leone, Giovanni Fiorillo, Franca Criscuoli, Stefano Ravasenghi, Laura Santagostini, Gelsomina Fico, Angela Spadafranca, Alberto Battezzati, Alberto Schiraldi, Federica Pozzi, Sara di Lello, Sandro Filippini, Simona Bertoli

**Affiliations:** 1 International Center for the Assessment of Nutritional Status (ICANS), University of Milan, Via Sandro Botticelli 21, 20133 Milan, Italy; E-Mails: giovanni.fiorillo@unimi.it (G.F.); franca.criscuoli@unimi.it (F.C.); stefano.ravasenghi@unimi.it (S.R.); angela.spadafranca@unimi.it (A.S.); alberto.battezzati@unimi.it (A.B.); simona.bertoli@unimi.it (S.B.); 2 Department of Food, Environmental and Nutritional Sciences (DeFENS), University of Milan, Via Celoria 2, 20133 Milan, Italy; E-Mail: alberto.schiraldi@unimi.it; 3 Department of Chemistry, University of Milan, Via Golgi 19, 20133 Milan, Italy; E-Mail: laura.santagostini@unimi.it; 4 Department of Pharmaceutical Sciences (DISFARM), University of Milan, Via Mangiagalli 25, 20133 Milan, Italy; E-Mail: gelsomina.fico@unimi.it; 5 Botanic Garden G.E. Ghirardi, Department of Pharmaceutical Sciences (DISFARM), University of Milan, Via Religione 25, 25088 Toscolano Maderno, Italy; 6 AVSI Foundation, Via Legnone, 20158 Milan, Italy; E-Mail: federica.pozzi@avsi.org; 7 Movimento Africa 70, Via Missori 14, 20900 Monza, Italy; E-Mail: sara.dilello@alice.it; 8 ACRA Foundation, Via Lazzaretto 3, 20124 Milan, Italy; E-Mail: sandro.filippini@acra.it

**Keywords:** *Moringa oleifera*, proximate analysis, antioxidant, flavonoids, phenolic acids, salicylic acid, ferulic acid, Chad, Sahrawi, Haiti

## Abstract

*Moringa oleifera* is a plant that grows in tropical and subtropical areas of the world. Its leaves are rich of nutrients and bioactive compounds. However, several differences are reported in the literature. In this article we performed a nutritional characterization and a phenolic profiling of *M. oleifera* leaves grown in Chad, Sahrawi refugee camps, and Haiti. In addition, we investigated the presence of salicylic and ferulic acids, two phenolic acids with pharmacological activity, whose presence in *M. oleifera* leaves has been scarcely investigated so far. Several differences were observed among the samples. Nevertheless, the leaves were rich in protein, minerals, and β-carotene. Quercetin and kaempferol glycosides were the main phenolic compounds identified in the methanolic extracts. Finally, salicylic and ferulic acids were found in a concentration range of 0.14–0.33 and 6.61–9.69 mg/100 g, respectively. In conclusion, we observed some differences in terms of nutrients and phenolic compounds in *M. oleifera* leaves grown in different countries. Nevertheless, these leaves are a good and economical source of nutrients for tropical and sub-tropical countries. Furthermore, *M. oleifera* leaves are a source of flavonoids and phenolic acids, among which salicylic and ferulic acids, and therefore they could be used as nutraceutical and functional ingredients.

## 1. Introduction

*Moringa oleifera* Lam. is a plant belonging to the monogeneric genus Moringa of the Moringaceae family. It is native of the Indian subcontinent, but, thanks to its ability to grow both in humid and hot dry lands and survive in less fertile soils chronically affected by drought, it has become naturalized in tropical and subtropical areas around the world [1]. It has been defined as a multi-purpose tree, as all parts of the plant are used for different purposes. Leaves are the most used part of the plant. In particular, they are used for human and animal nutrition and in the traditional medicine for the treatment of many ailments [2]. Several *in vitro* and *in vivo* studies, indeed, have ascribed numerous pharmacological properties to *M. oleifera* leaves, although the scientific evidence on human beings is still limited [3]. The leaves can be also used as a natural plant growth enhancer and as biopesticide [4].

Leaves are rich in proteins, calcium, iron, potassium, vitamins (particularly C and E), β-carotene [5,6,7,8,9,10,11], and in antioxidant and bioactive compounds, such as flavonoids, phenolic acids, glucosinolates and isothiocanates, tannins and saponins [1,3], first responsible of the numerous pharmacological properties ascribed to *M. oleifera* leaves [3]. These features make the plant suitable to be used in the fight against malnutrition and as medicinal plant in underdeveloped and developing countries.

Even though several worldwide studies report the nutritional characteristics and the total phenolic content of *M. oleifera* leaves, few studies have investigated their phenolic profile [12,13]. Moreover, these findings are often controversial. Several factors, such as the different agro-climatic conditions existing among the countries where the plant grows, genetic factors, the different cultivation techniques, and the drying method used [14,15,16,17], can affect the content of nutrients and phenolic compounds. Forster *et al.* [16] suggest that because of an enormous suggestibility of secondary metabolites by the environment, findings should be proven in field trials in *M. oleifera* cultivation areas.

Lastly, the content of some compounds with documented pharmacological activity, such as salicylic and ferulic acids [18,19,20,21], has been scarcely investigated so far. Salicylic acid is mainly known as the principal metabolite and active component of aspirin, an anti-inflammatory drug, and seems to be involved in the prevention of some types of cancer [18]. Ferulic acid possesses various physiological properties, such as inhibition of tumor promotion, reduction of serum and hepatic lipid profiles (mainly total cholesterol), reduction of the triglycerides level, sustaining a protective action against liver injury [22]. However, their content in *M. oleifera* leaves has been little investigated so far.

In this article, we have performed a nutritional characterization and a phenolic profiling of *M. oleifera* leaves collected in Haiti, Chad, and Southwestern Algeria, where the Sahrawi refugee camps have been located since 1975; three areas in which *M. oleifera* leaves are used by the local population, but whose nutritional characteristics and phenolic profile are not available. These three countries are partially or totally dependent on humanitarian aid for the supply of food and medicines. The use of *M. oleifera* locally-grown both for nutritional and pharmacological purposes, could give these countries greater independence from humanitarian aid. However, in light of previous considerations about the effects of several factors affecting the composition of the leaves, before using *M. oleifera* leaves for nutritional and pharmacological purposes, a characterization of the leaves needs to be performed. The third, and last, aim of the present study was to investigate the presence of salicylic and ferulic acids in *M. oleifera* leaves.

## 2. Results and Discussion

Firstly, we performed a nutritional characterization of the leaves collected in different countries (Table 1). Residual moistures of 7.3 ± 0.5, 6.0 ± 0.2, and 8.8 ± 0.1 g/100 g were found in the leaves collected in Chad, Sahrawi camps, and Haiti, respectively. As expected, the leaves presented a high content of crude protein. The Haitian leaves were the poorest of protein, but similar amounts were found in the leaves collected in Mexico, Niger, and Thailand [5,8,10]. Crude fat content was moderate, in agreement with some previous studies [6,8,12], but lower [9,10] or higher [5] than that found by other studies. Fiber content was very high, especially in the insoluble fraction. The Haitian leaves were the richest in fiber. These amounts are similar to the fiber content of *M. oleifera* leaves collected in Mexico [8], but greater than amounts found in the leaves collected in other countries [5,9]. Sugar content was very different among the samples. Sucrose content was higher in the leaves collected in the Sahrawi camps, while the leaves collected in Haiti were the richest in glucose and fructose. Maltose was found to be not-detectable in all samples. This is the first report in which sugar content has been investigated. *M. oleifera* leaves are a good source of dietary minerals. The predominant minerals in the leaves were calcium and magnesium. The leaves collected in the Sahrawi camps were found the richest in calcium, iron, sodium, and copper, while magnesium and zinc contents were similar among the samples. Mineral contents reported in the literature are very different. The calcium content in the current study was within the range reported in the literature [5,6,9,10,11,12]. In our samples, the magnesium content was similar to the amounts found in the leaves collected in South Africa [6] and Ghana [7,12], but over five-fold greater than amounts found in the leaves collected in Pakistan [11]. The sodium content found in the leaves collected in Haiti and Chad was similar to the amounts reported by some previous studies [10,11], while the sodium content found in the leaves collected in the Sahrawi camps was the greatest. It should be noted that other studies found lower [12] or not detectable [9] amounts of sodium in the leaves from other countries. The iron content found in the leaves collected in the Sahrawi camps was in agreement with the amounts found in the leaves collected in some provinces of Pakistan and Thailand [5,11], while the iron content of the leaves collected in Haiti and Chad was about three- to four-fold lower. Finally, the amounts of zinc and copper found in our samples were in agreement with previous investigations [10,11].

**Table 1:** Nutritional characterization of *M. oleifera* leaves from Chad, Sahrawi refugee camps (Southwester Algeria), and Haiti, expressed as dry matter.

Nutrients		CHAD	SAHRAWI CAMPS	HAITI
		Mean ± sd	Mean ± sd	Mean ± sd
Proteins	g/100 g	31.47 ± 0.12	27.98 ± 0.12	20.80 ± 0.01
Lipids	g/100 g	6.65 ± 0.28	4.85 ± 0.30	7.05 ± 0.11
Total fibre	g/100 g	33.29 ± 0.63	31.88 ± 0.34	37.63 ± 1.00
Insoluble fibre	g/100 g	23.97 ± 0.46	27.94 ± 0.27	30.09 ± 1.40
Soluble fibre	g/100 g	9.31 ± 0.18	3.94 ± 0.07	7.54 ± 0.40
Starch (*estimated by difference*)	g/100 g	12.41 ± 0.52	11.37 ± 0.06	13.75 ± 0.31
Glucose	g/100 g	2.41 ± 0.20	2.03 ± 0.17	4.57 ± 0.16
Fructose	g/100 g	0.47 ± 0.07	0.54 ± 0.04	4.81 ± 0.31
Sucrose	g/100 g	2.50 ± 0.08	7.96 ± 0.00	1.77 ± 0.08
Maltose	g/100 g	ND	ND	ND
Ashes	g/100 g	10.79 ± 0.01	13.38 ± 0.05	9.62 ± 0.02
Sodium	mg/100 g	307.65 ± 1.49	791.28 ± 4.43	262.50 ± 5.45
Calcium	mg/100 g	1839.10 ± 12.82	2743.38 ± 39.69	2150.26 ± 56.07
Iron	mg/100 g	17.03 ± 0.79	41.68 ± 1.08	11.91 ± 0.82
Zinc	mg/100 g	2.48 ± 0.01	3.09 ± 0.01	2.18 ± 0.06
Magnesium	mg/100 g	562.49 ± 9.07	489.94 ± 8.76	533.51 ± 23.87
Copper	mg/100 g	ND	1.22 ± 0.08	0.66 ± 0.00
Phytates	g/100 g	2.95 ± 0.02	3.03 ± 0.15	2.55 ± 0.19
β-carotene	mg/100 g	19.03 ± 0.19	28.53 ± 1.71	10.01 ± 0.07

Abbreviation: ND = not detectable.

Although the mineral content of the leaves of *M. oleifera* was high, the phytate content was equally high. The amounts found in the leaves are in agreement with previous investigations [23,24,25] and are greater than those found in legumes and cereals [26], but slightly lower compared to the amounts found in wheat bran [27]. Unfortunately, these compounds are able to bind minerals, making them unavailable for intestinal absorption. Future researches aimed to identify ways to reduce the phytate content of *M. oleifera* leaves are recommended. Leaves were found rich in β-carotene, more than orange, carrots, and melon, primary vegetable sources of this compound [28]. The leaves collected in the Sahrawi camps were the richest of β-carotene. The amounts found in the leaves collected in Chad and Sahrawi camps were similar to the amount found in the leaves collected in South Africa [6], but lower compared to the amounts found in India [14], whereas the β-carotene content of the leaves collected in Haiti was lower than the amounts reported in the literature [3].

Secondly, we evaluated the total antioxidant activity of the leaves, and determined their contents of total polyphenols and salicylic and ferulic acids (Table 2). In addition, we performed a LC-MS analysis of leaf methanolic extract in order to provide a picture of phenolic compounds present in plants grown in these three countries.

**Table 2:** Bioactive compounds content of *M. oleifera* leaves grown in Chad, Sahrawi refugee camps (Southwestern Algeria), and Haiti, expressed as dry matter.

Compounds		CHAD	SAHRAWI CAMPS	HAITI
		Mean ± sd	Mean ± sd	Mean ± sd
TEAC	µmol trolox/g	304.63 ± 8.70	427.16 ± 33.94	335.61 ± 7.37
Total polyphenols	mg/100 g	2813 ± 51	3552 ± 388	2545 ± 194
Salicylic acid	mg/100 g	0.14 ± 0.02	0.20 ± 0.01	0.33 ± 0.04
Ferulic acid	mg/100 g	6.61 ± 0.15	8.86 ± 0.18	9.69 ± 0.26

*M. oleifera* leaves presented a high total antioxidant capacity, consequently to a high content of total polyphenols. In particular, the leaves collected in Sahrawi camps presented the highest total antioxidant activity and were found the richest of total polyphenols, while the leaves collected in Chad and Haiti were similar to each other. The total antioxidant activity found in our sample was, however, lower than that reported by Pari *et al.* [29]. On the other hand, the total polyphenol content was in agreement with some previous investigations [30,31,32], but lower than amounts reported by others [15,33].

**Figure 1:**
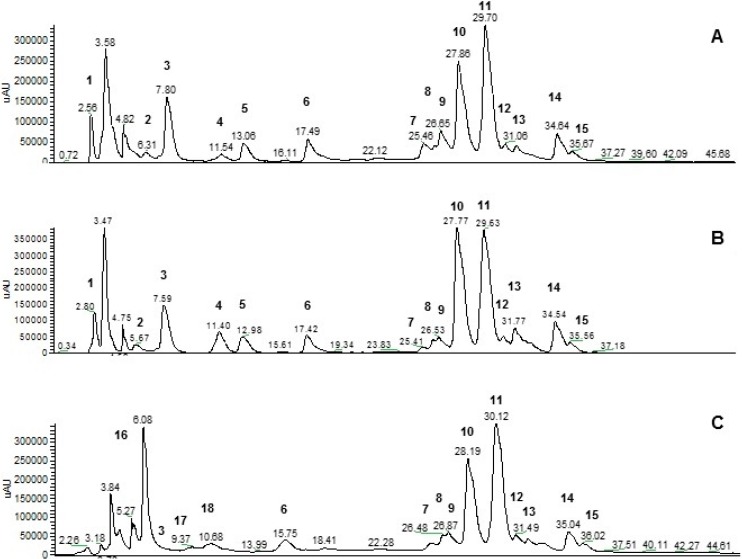
HPLC chromatogram of methanolic extracts of *M. oleifera* leaves collected in Chad (**A**); Sahrawi camps (**B**); and Haiti (**C**).

In Figure 1 the HPLC-UV chromatogram of the methanolic extracts of *M. oleifera* leaves collected in the three countries are shown. As expected, flavonoids were the principal phenolic compounds. Quercetin-3-*O*-glucoside (compound **10**), quercetin-3-*O*-(6′′-malonyl)glucoside (compound **11**), quercetin-3-*O*-(X′′-malonyl)glucoside (compound **12**), kaempferol-3-*O*-glucoside (compound **13**), and kaempferol-3-*O*-malonylglucoside (compound **14**) were identified in all samples in agreement with previous reports [12,13,16]. In addition, we have attributed peaks at about 17.42 (17.49), 26.13 (26.65), and 35.56 (35.67) min respectively to a glicosilated flavonoid deriving from condensation of kaempferol with a rhamnose and a glucose (compound **6**), quercetin-3-*O*-glucoside-7-*O*-rhamnoside (compound **8**), and to methyl-*O*-quercetin-malonylglucoside (compound **15**). Interestingly the leaves collected in Chad and Sahrawi refugee camps were characterized by a isorhamnetin-rhamnosyl-glucoside (13.0 min) (compound **5**) and a diglucoside, probably composed by quercetin and two rhamnose units, or by kaempferol condensed to a rhamnosyl-glucoside (compound **4**). This study also confirms the presence of apigenin-*C*-glucoside [34] (compound **7**) and add that apigenin-*O*-glucoside (compound **9**) is present as well. On the basis of ESI (+)-MS and UV-VIS spectra, in addition to flavonoids in all extracts, the peak at about 6.98–7.6 min was identified as hexadecylferulate (compound **3**). Moreover, we attributed other compounds, in which presence in *M. oleifera* leaves was not yet reported. In the leaves collected in Chad and Sahrawi camps, the compound eluting at 2.56 min (2.80 min in Sahrawi sample) was identified as 5-hydroxyferulic acid glucoside (compound **1**), a precursor of synaptic acid, while the peak at about 6.31 min (5.67 min in Sahrawi) was attributed to diferuloyl-(5OH-feruloyl)spermidine (compound **2**). Differently, in the methanolic extract of Haitian *M. oleifera* leaves, compounds eluted in the range 3.84–6.08 and respectively at 9.37 and 18.41 min were identified as glucosinolates (compounds **16**), dicoumaroyl putrescine (compound **17**), and di(dihydrocaffeoyl)spermidine (compound **18**), two other polyamine alkaloids.

Finally, all of our samples presented detectable amounts of salicylic and ferulic acids. Leaves collected in Haiti were the richest in both salicylic and ferulic acids. This is the first study that reports the presence of salicylic acid in *M. oleifera* leaves. Salicylic acid concentration found in *M. oleifera* leaves was comparable with amounts presented in nectarine, pineapple, tomato, and asparagus, but higher than amounts found in other fruit and vegetables [35]. Only spices present a concentration of salicylic acid 10-fold higher [35]. Similarly, ferulic acid amounts found in *M. oleifera* leaves were comparable with the amounts found in several fruits and vegetables, such as orange, eggplant, spinach, red cabbage, and peanuts, but widely lower than amounts found in cereals [21].

As expected, leaves collected in these three countries presented differences both in the nutritional composition and in the quantitative phenolic content, as a consequence of different environmental conditions, genetic factors, cultivation, and drying techniques. Forster *et al.* [16] report high ecotype variability in the secondary metabolite content in *M. oleifera* leaves. In addition, the authors observed that water deficiency determined an increment of phenolic compound production. Water stress, indeed, determined an increase of oxidative stress that plants counterattack, increasing the production of antioxidant compounds [36]. Furthermore, water stress also determines an increment of osmotic stress that plants counterattack, increasing the accumulation of proline, sucrose, and ions (K^+^, Na^+^ and Cl^−^) [36]. Water deficiency could be one of reasons why leaves collected in the Sahrawi camps presented a different nutritional characterization and a different total antioxidant ability. Sahrawi refugee camps, indeed, are located in the Southwestern Algeria, in the Sahara desert, where water availability is very low. However, we cannot exclude the existence of different ecotypes of *M. oleifera* in these three countries, especially between African countries and Haiti. Definitely, these findings, in agreement with other authors, reinforce the need to know the nutritional and phenolic characteristics of *M. oleifera* leaves grown locally before using them for nutritional and pharmacological purposes.

Regardless of the differences found in the leaves of *M. oleifera* collected in different countries, these findings are of particular relevance for sub-Saharan and Caribbean populations, who suffer malnutrition. Last reports show a high prevalence of undernutrition, especially among children, in these three areas. Specifically, the prevalence of stunting, wasting, and underweight are 39%, 16%, and 30% in Chad [37], 29.1%, 9.1%, and 18.6% in the Sahrawi refugee camps [38], and 22.2%, 4.3%, and 10.5% in Haiti [39]. The main causes of child malnutrition in these countries include poor maternal nutrition, poor hygienic practices, poor feeding practices, and food quality, which lead to a deficiency of essential nutrients. Principal nutrient deficiency concerns proteins, vitamin A, iron, zinc, calcium, and other micronutrients [40]. *M. oleifera* leaves, thanks to their high protein and essential amino acids (44%) [8], minerals, and β-carotene contents, could represent an economical source of all these nutrients. Moreover, thanks to their contribution in bioactive compounds (flavonoids and phenolic acids, among which salicylic and ferulic acids), *M. oleifera* leaves could both compensate the low intake of phenolic compounds due to the scarce fruit and vegetable consumption observed in these populations [41] and be appealed for a potential exploitation in Western countries as nutraceutical and functional ingredients.

Particular attention should be given to the effects of cooking. *M. oleifera* leaves can be eaten raw, but, generally, they are eaten cooked, added to local food preparations or to prepare decoctions [3]. Unfortunately, cooking could cause a depletion of nutrients and bioactive compounds. The magnitude of the losses depends on the temperature and the duration of treatment. Therefore, in order to avoid excessive loss of nutrients and bioactive compounds, the leaves of *M. oleifera* may be added at the end of cooking or to preparations already cooked. Future research aimed to measure the nutritional and phenolic losses caused by cooking will be useful to determine the magnitude of such losses and will fill this lack of information. Finally, future dietary interventions, aimed to involve *M. oleifera* leaves as a nutritional supplement, should take into account such losses caused by cooking.

## 3. Experimental Section

### 3.1. Samples

Fresh leaves were collected from different plants of *M. oleifera* spontaneously grown in Chad, Sahrawi refugee camps, and Haiti. Samples from Chad were collected in Logone’s Valley, in the south of the country, next to the border with Cameroon. Samples from Sahrawi refugee camps were collected in the Tindouf Province, in Southwestern Algeria, next to the border with Morocco. Finally, samples from Haiti were collected in the area around Port-au-Prince. Plants have been identified by Dr. Alberto Spada, assistant professor of Botany at University of Milan. The collection was carried out between March 2011 and November 2012. After collecting, the leaves were dried at room temperature and 200 g of each dried sample were packaged and sent to our laboratory. Upon arrival, dried leaves were ground to a fine powder with an electric grinder and stored at −80 °C.

Proximate analysis, trolox equivalent antioxidant capacity (TEAC), total phenols, and SA and FA contents and phenolic profile were experimentally determined. All analyses were performed in triplicate by the same operators.

### 3.2. Chemicals

Sodium hydroxide, anhydrous sodium sulphate, glucose, fructose, sucrose, ABTS, potassium persulfate, α-amylase, amyloglucosidase, protease, ethanol, iron(III) chloride hexahydrate, sulfosalicylic acid, and trolox were purchased from Sigma-Aldrich (St. Louis, MO, USA). Boric acid, mixed indicator for ammonia titrations, Folin-Ciocalteu reagent, sodium carbonate, tetrahydrofuran, methanol, dichloromethane, sodium chloride, anhydrous monobasic sodium phosphate, disodium phosphate, hydrochloric acid, formic acid, petroleum ether, acetonitrile, ethyl acetate, and standards of Na, Ca, Fe, Zn, and Mg were purchased from Merck (Dramstadt, Germany). Diethyl ether, petroleum ether, acetone, sulfuric acid, methanol, and acetonitrile were purchased from VWR BDH Prolabo (Radnor, PA, USA). Kjeldahl catalyst, sodium hydroxide solution for determination of sugars, and gallic acid were purchased from Fluka (St. Louis, MO, USA). *N*,*O*-bis(trimethylsilyl)trifluoroacetamide, and trimethyl-chlorosilane were purchased from Supelco (Bellafonte, PA, USA). d4SA were purchased from CIL (Tewksbury, MA, USA). Finally, d3FA was purchased from CDN Isotopes (Pointe-Claire, QC, Canada).

### 3.3. Proximate Analysis

The moisture content was determined by drying the samples at 105 °C for 6 h. The ash content was determined by incineration of the samples at 550 °C for 6 h. The mineral content was determined by atomic absorption spectrophotometry. Kjeldahl and Soxlet methods were used to determine protein and lipid content respectively [42]. Prosky’s method was used to determine the soluble and insoluble fiber content [43]. Glucose, fructose and sucrose contents were determined by means of anion exchange HPLC with pulsed amperometric detection [44]. 1 g of sample was extracted with 200 mL of distilled water and, after 1 h at 60 °C, the solution was filtered through Whatman filter 0.45 μm. The filtrate was appropriately diluted to reduce the sugar concentration within 0.5–5 ppm range. 20 μL of extract solution were injected into the HPLC column. The HPLC system was equipped with an autoinjector LC 717 Plus (Waters, Milford, MA, USA), a CarboPac PA1 (4 × 250 mm) plus guard column CarboPac PA1 Guard (4 × 50 mm) (Dionex, Sunnyvale, CA, USA), a Spectra System LC P 1000 isocratic pump (Thermo Fisher Scientific Inc., Waltham, MA, USA) and a pulsed amperometric detector ED50 (Dionex, Sunnyvale, CA, USA). NaOH 160 mM with a flow rate of 1 mL/min, was used as mobile phase. Pulsed amperometric detection was carried out with the following pulse potentials and durations: E_OX_ = +0.6 V (t_OX_ = 430 ms), E_DET_ = +0.1 V (t_DET_ = 400 ms), and E_RED_ = −2.0 V (t_RED_ = 420 ms). Standard solutions of each sugar were prepared to encompass the 0.5–5 ppm concentration range. 20 μL of each sugar solution was injected in HPLC system to draw the values for the calibration curve.

### 3.4. Determination of β-Carotene

The β-carotene content was obtained with the method described by Panfili *et al.* [45]. 5 g of sample were extracted with 40 mL of tetrahydrofuran (THF + BHT 1 g/L). The mixture was shaken vigorously for 15 min. The solution was centrifuged at 400× *g* for 1 min and the organic phase was separated. The residual phase was extracted again with tetrahydrofuran. The two organic phases were combined in a separation funnel and 40 mL of dichloromethane and 40 mL of solution of sodium chloride were added in order to purify the solution. The solution was evaporated in a rotavapor at 35 °C and reconstituted with a mixture of methanol and tetrahydrofuran (MetOH/THF 95:5). It was filtered on Whatman filter (PTFE, porosity 0.45 μM) and 20 μL were injected into the HPLC system. The HPLC system was equipped with vydac 201TP54 column (Alltech Italia Srl, Milan, Italy), a binary pump LC 1525 (Waters, Milford, MA, USA), and a photodiode array detector 2996 (Waters, Milford, MA, USA). The mobile phase was a solution of acetonitrile, methanol, and dichloromethane (84%/15%/1% *v*/*v*) with a flow rate of 1.2 mL/min. Spectrophotometric detection was performed at 450 nm. Standard solutions of β-carotene were prepared to encompass the 0.5–5 μg/mL concentration range. For each standard solution the absorbance determined at 450 nm was used to draw the calibration curve.

### 3.5. Determination of Phytates

Phytates content was determined with Vaintraub and Lapteva method [46] with modification proposed by Gao *et al.* [47] Briefly, 0.5 g of sample were stirred in 10 mL 2.4% HCI for overnight. The contents were centrifuged at 1000× *g* for 20 min at 10 °C. The supernatant was recovered and added to 1× *g* NaCl. The contents were shaken at 350 rpm for 20 min to dissolve the salt and were allowed to settle at −20 °C for 20 min. The mixtures were centrifuged at 1000× *g* at 10 °C for 20 min and the clear supernatant was collected. 60 μL of the clear supernatant were stirred with 1440 μL of H_2_O. 500 μL of modified Wade reagent (0.03% FeCl_3_∙6H_2_O + 0.3% sulfosalicylic acid) were added to the sample, thoroughly mixed on a vortex, and centrifuged at 1000× *g* at 10 °C for 10 min. 1 mL of solution were transferred in a cuvette and the absorbance at 500 nm was measured with a spectrophotometer Cary 5E (Varian, Palo Alto, CA, USA). A series of calibration standards containing 0, 0.015, 0.045, 0.09, 0.15 and 0.3 g/L PA-P were prepared from sodium phytate, the P content of which was established as 18.38%. The calculation of sample PA-P content followed the method described in Latta and Eskin [48].

### 3.6. Determination of TEAC

Total antioxidant capacities were measured with the Serpen’s method [49]. ABTS solution was prepared dissolving 38.4 mg of ABTS in 5 mL of deionized water, and potassium persulfate solution was prepared dissolving 6.6 mg of potassium persulfate in 5 mL of deionized water. A total of 10 mL of ABTS radical regent (ABTS^●+^) was obtained mixing the two solutions. ABTS^●+^ was kept in a dark at room temperature for 12 h. After this time, 10 mL of ABTS^●+^ solution were diluted in 800 mL of a water/ethanol (50:50 *v*/*v*) in order to obtain a working standard solution of ABTS^●+^ with an absorbance of 0.75–0.80 at 734 nm. 5 mg of sample were added to 100 mL of ABTS^●+^ working solution. The tube was vortexed for 2 min and placed on an orbital shaker at 400 rpm for 30 min at room temperature. After this time, the tube was centrifuged for 2 min at 9200× *g*, the supernatant was collected and left for 30 min at room temperature. 2 mL of supernatant was transferred in a cuvette and the absorbance at 734 nm was measured by spectrophotometer Cary 5E (Varian, Palo Alto, CA, USA). The inhibition percentage of the sample was calculated. Trolox was used as a reference and standard solutions were prepared to encompass the 0–600 μg/mL range. A 0.1 mL of each trolox solution was added to 9.9 mL of ABTS^●+^ and, after 30 min of incubation at room temperature, the absorbance at 734 nm was measured. For each standard solution the percentage of inhibition of trolox was calculated and the calibration curve for ABTS was obtained. TEAC was expressed as µmol Trolox equivalent/g of sample.

### 3.7. Determination of Total Phenols

The total phenolic content was determined with the Folin-Ciocalteu method [50] on extracts obtained according to the instructions reported by Naczk & Shahidi [51]. 0.5 g of Moringa leaves were added to 10 mL of aqueous methanol (80%) and placed in a stirred bath for 2 h at room temperature. After the extraction phase, 2.5 mL of previously diluted (1:10) Folin-Ciocalteu reagent and to 2.0 mL of solution of sodium carbonate were added to 0.5 mL of methanolic extract. The solution obtained was placed in a water bath for 5 min at 50 °C and subsequently cooled with ice. 0.1 mL of solution were transferred in a cuvette and the absorbance at 760 nm was measured with a spectrophotometer Cary 5E (Varian, Palo Alto, CA, USA). Gallic acid was used as a reference and standard solutions were prepared to encompass the 0–50 μg/mL concentration range. For each standard solution the absorbance at 760 nm was measured and the calibration curve was obtained. The total phenolic content was expressed as mg gallic acid equivalent/100 g.

### 3.8. Extraction and HPLC Analysis of Polyphenols

1 g of dried leaves was subjected to extraction with a sequence of solvents at increasing polarity (petroleum ether 40–60, dichloromethane and methanol). Extraction was performed six times for each solvent with equal volumes and timing (20 mL, 1 h), leading to three organic fractions containing respectively lipids, pigments and polyphenols.

The dry sample obtained with methanol during the extraction procedure described above was redissolved in the same solvent, diluted in acetonitrile and then injected. HPLC analyses were performed at room temperature on a Jasco HPLC equipped with PDA detector and Lichrocart^®^ RP-18 column (250 × 4.6 mm, 3 µm, Merck KGaA, Darmstadt, Germany). Eluent composition was varied between 0.1% formic acid in water (A) and pure acetonitrile (B) and applying the following gradient program: 0–2 min B 10%, 2–30 min B 10%–25%, 30–40 min B 25%–30%, 40–45 min B 30%, 45–47 min B 30%–10%, at a flow rate of 0.6 mL·min^−1^. LC-MS analyses were performed on a Thermo Finnigan LC-MS system, equipped with a PDA detector and a LCQ Advantage mass spectrometer, using the same column and gradient conditions as reported previously. Results are reported as integrated areas.

### 3.9. Extraction and GC-MS Determination of Salicylic and Ferulic Acids Contents

The contents of total salicylic and ferulic acids were determined using a reference method [52] with some modifications [19]. Briefly, 200 mg of sample were mixed with 5 mL of NaOH 1 M and left at room temperature overnight. The extracts were shaken for 1 h and acidified to pH 1–2 with HCl 6 N. It was eventually transferred into a separation funnel and extracted twice with 15 mL of ethyl acetate [53]. The organic solutions were filtrated through anhydrous Na_2_SO_4_. The extract was diluted up to prefixed volume in a volumetric flask with ethyl acetate. 100 μL of internal standard mix solution (500 ng/mL) contenting d4-SA and d3-FA was added to an aliquot of 1 mL of the extract and dried with stream of nitrogen. The dried samples were derivatized by adding 50 μL of a solution (1:1) of acetonitrile and *N*,*O*-bis(trimethylsilyl)trifluoroacetamide with 1% trimethylchlorosilane and keeping the solution at 70 °C for 1 h.

**Figure 2:**
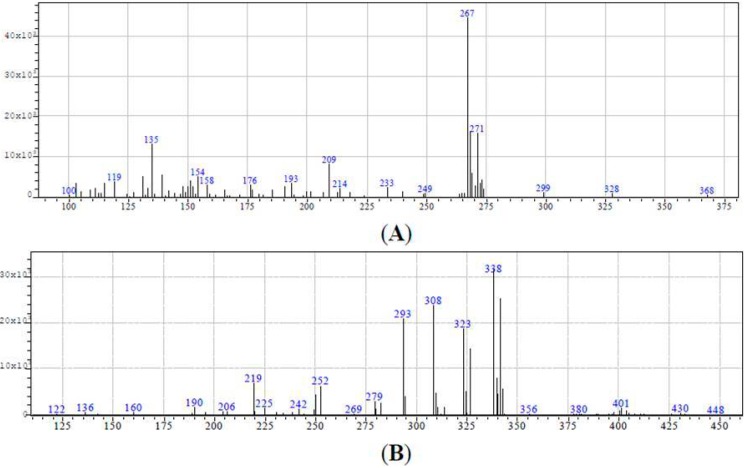
Mass spectra of salicylic (**A**); and ferulic (**B**) acids with internal standards.

The analytical quantification of salicylic and ferulic acids was performed by isotope-dilution Gas Chromatography-Mass Spectrometry (GC-MS), by means of a gas chromatograph GC-17A (Shimadzu, Tokyo, Japan) interfaced with a single-quadrupole mass spectrometer MS-QP5050 (Shimadzu, Tokyo, Japan). Gas chromatography separation was performed on a DB-5-MS capillary column (30 m; 0.25 mm i.d., 0.25 µm film thickness) (J&W Scientific, Folsom, CA, USA). The mass spectrometer was operated in electron impact (70 eV) mode and the equipment was set up to carry out selective ion monitoring (SIM). The qualitative analysis was performed analyzing the range 50–450 U of individual standards. The mass spectra of salicylic and ferulic acids with internal standards derivatives are shown in Figure 2. Selective ion monitoring for each analyte were 267 *m*/*z* for salicylic acid and 271 *m*/*z* for its internal standard and 323 *m*/*z* for ferulic acid and 326 for its internal standard. Standard solutions of salicylic and ferulic acids were made at a concentration range between 10–100 ng/mL. Identification of derivatized salicylic acid was achieved by comparing the gas chromatographic retention times and the mass spectra of the extracts with those of the authentic standards. In each sample, the quantification of salicylic and ferulic acids was performed using calibration curves obtained by measuring the response ratio of each derivative to that of the internal standard, and were plotted *vs.* their known concentrations. Standard curves were linear for all individual compounds in the concentration range investigated, with the correlation coefficients *r* > 0.999. The limits of detection and quantification were 0.6 and 2 ng for salicylic acid and 0.5 and 1.8 ng for ferulic acid.

### 3.10. Statistical Analysis

All analyses were performed using Excel 2007. The relevant results were presented as mean ± sd of three replicas.

## 4. Conclusions

In conclusion, we observed some differences in terms of nutrients and phenolic compounds in the leaves of *M. oleifera* grown in different countries. Nevertheless, *M. oleifera* leaves are a good and economical source of nutrients and, therefore, they are a promising food supplement to contrast the malnutrition (especially the chronic malnutrition of children during the first 1000 days of the life) that is endemic in many populations of tropical and sub-tropical areas. Furthermore, *M. oleifera* leaves are a source of flavonoids and phenolic acids, among which salicylic and ferulic acids and, therefore, they could be used as nutraceutical and functional ingredient. 
